# Hemoglobin Cleavage Site-Specificity of the *Plasmodium falciparum* Cysteine Proteases Falcipain-2 and Falcipain-3

**DOI:** 10.1371/journal.pone.0005156

**Published:** 2009-04-09

**Authors:** Shoba Subramanian, Markus Hardt, Youngchool Choe, Richard K. Niles, Eric B. Johansen, Jennifer Legac, Jiri Gut, Iain D. Kerr, Charles S. Craik, Philip J. Rosenthal

**Affiliations:** 1 Department of Medicine, Division of Infectious Diseases, University of California San Francisco, San Francisco, California, United States of America; 2 Department of Cell and Tissue Biology, University of California San Francisco, San Francisco, California, United States of America; 3 Department of Pharmaceutical Chemistry, University of California San Francisco, San Francisco, California, United States of America; 4 Department of Cellular and Molecular Pharmacology, University of California San Francisco, San Francisco, California, United States of America; Federal University of São Paulo, Brazil

## Abstract

The *Plasmodium falciparum* cysteine proteases falcipain-2 and falcipain-3 degrade host hemoglobin to provide free amino acids for parasite protein synthesis. Hemoglobin hydrolysis has been described as an ordered process initiated by aspartic proteases, but cysteine protease inhibitors completely block the process, suggesting that cysteine proteases can also initiate hemoglobin hydrolysis. To characterize the specific roles of falcipains, we used three approaches. First, using random P_1_ – P_4_ amino acid substrate libraries, falcipain-2 and falcipain-3 demonstrated strong preference for cleavage sites with Leu at the P_2_ position. Second, with overlapping peptides spanning α and β globin and proteolysis-dependent ^18^O labeling, hydrolysis was seen at many cleavage sites. Third, with intact hemoglobin, numerous cleavage products were identified. Our results suggest that hemoglobin hydrolysis by malaria parasites is not a highly ordered process, but rather proceeds with rapid cleavage by falcipains at multiple sites. However, falcipain-2 and falcipain-3 show strong specificity for P_2_ Leu in small peptide substrates, in agreement with the specificity in optimized small molecule inhibitors that was identified previously. These results are consistent with a principal role of falcipain-2 and falcipain-3 in the hydrolysis of hemoglobin by *P. falciparum* and with the possibility of developing small molecule inhibitors with optimized specificity as antimalarial agents.

## Introduction

Malaria, especially that caused by *Plasmodium falciparum*, is responsible for hundreds of millions of episodes of disease, and probably at least one million deaths each year [1], [2]. Among impediments to effective control of this disease is increasing resistance to most available drugs [3]. New artemisinin-based combination therapies offer excellent efficacy, but resistance to most artemisinin partner drugs has already been seen [4], and resistance to artemisinins may be emerging [5]. The pipeline for development of other classes of effective antimalarials, especially compounds that act against novel biochemical targets, is limited. To facilitate development of such compounds, it is important to characterize the biochemical features and biological roles of new drug targets.

Falcipain-2 and falcipain-3 are papain-family cysteine proteases of *P. falciparum* that are currently the subjects of in-depth drug discovery efforts [6]. These proteases are expressed sequentially across the life cycle of erythrocytic parasites, with expression of falcipain-2 beginning in early trophozoites followed by expression of falcipain-3 in late trophozoites and schizonts [7]. The proteases are hemoglobinases that reside in the food vacuole of intraerythrocytic parasites, where they degrade hemoglobin, in concert with other proteases [8], to provide the parasite with amino acids for growth and development [9]. Treatment of erythrocytic parasites with cysteine protease inhibitors or disruption of the falcipain-2 gene results in accumulation of undegraded hemoglobin in the food vacuole, confirming a role for this enzyme in hemoglobin hydrolysis [6], [10]. Disruption of the falcipain-3 gene could not be achieved, but the gene was readily replaced with a tagged functional copy, indicating that falcipain-3 is essential for survival of intraerythrocytic parasites [11]. These results support studies of falcipain inhibitors as new antimalarial agents.

The hydrolysis of hemoglobin by malaria parasites is a massive proteolytic enterprise, which appears to be responsible for the degradation of most erythrocytic hemoglobin over the course of a 48 hour developmental cycle [8], [12]. It has been described as an ordered pathway, with initial cleavage by aspartic proteases followed by action of other proteases, eventually leading to free amino acids [12]. However, falcipain inhibitors completely block hemoglobin hydrolysis by erythrocytic parasites [13], and falcipain-2 and falcipain-3 hydrolyze native hemoglobin under the biochemical conditions of the food vacuole [14], suggesting that falcipains also initiate hemoglobin hydrolysis in *P. falciparum*. To better characterize the roles of falcipain-2 and falcipain-3, we evaluated activities of these proteases against a library of small peptide substrates, a series of larger peptides spanning the sequences of α and β globin, and intact human hemoglobin. Falcipain-2 and falcipain-3 demonstrated a marked preference for cleavage of small peptide substrates with P_2_ Leu, but the enzymes showed less specificity against larger peptides and intact hemoglobin, with hydrolysis at multiple sites. These results suggest that hemoglobin hydrolysis by *P. falciparum* is not a highly ordered process, but rather that falcipain-2 and falcipain-3 rapidly cleave hemoglobin at multiple sites to facilitate rapid hydrolysis of this substrate.

## Methods

### Synthesis of Recombinant Falcipains

Expression, solubilization and refolding of falcipain-2 and falcipain-3 were performed as described elsewhere, with slight modifications [15], [16], [17]. In brief, urea solubilized inclusion bodies from bacteria over-expressing the mature domains of either falcipain-2 or falcipain-3 fused to a 6×HIS tag were purified by ultra-filtration and bound to Nickel-Nitrilotriacetic acid (Ni-NTA) columns. After several washes with buffer containing 8 M urea and 10–60 mM imidazole, bound protein was eluted sequentially with 500 mM and 1 M imidazole in the presence of urea. Refolding of falcipain-2 was achieved by diluting the eluates 100-fold in 100 mM Tris-HCl, 30% glycerol, 250 mM arginine, 1 mM EDTA, 1 mM reduced-glutathione (GSH), 1 mM oxidized-glutathione (GSSG), pH 9.2 and incubating at 4°C overnight. Refolding of falcipain-3 was achieved by diluting the eluates 100-fold in 100 mM Tris-HCl, 15% sucrose, 250 mM arginine, 1 mM EDTA, 1 mM GSH, 1 mM GSSG, pH 9.2 and incubating at 4°C overnight. The refolded proteins were concentrated 50-fold, run over a Q-Sepharose column, and eluted with a gradient of NaCl starting at 500 mM. Eluates were examined by SDS-PAGE and for activity against the synthetic substrate, Z-Leu-Arg-AMC (benzyloxycarbonyl-Leu-Arg-7-amino-4-methyl-coumarin). Fractions with highest enzyme activity were pooled and concentrated using a 10 KDa cut-off ultra-filtration column (Amicon). Enzymes were stored in 50% glycerol at −80°C.

### P_1_- P_4_ Specificity Determination Using Tetrapeptide Scanning Libraries

To determine the P_1_- P_4_ specificity of falcipain-2 and falcipain-3, complete diverse positional scanning substrate libraries were used [18]. The libraries were composed of peptide-conjugated ACC (7-amino-4-carbamoylmethylcoumarin) fluorophore substrates and contained 160,000 different P_1_- P_4_ peptide sequences. In the P_1_-, P_2_-, P_3_-, and P_4_- libraries, the P_1_-_4_ positions were spatially addressed with 20 amino acids (cysteine was replaced with norleucine); whereas the remaining three positions were randomized with equimolar mixtures of 20 amino acids (cysteine was replaced with norleucine). The cleavage of the substrates by a protease released free ACC, resulting in a shift of the excitation and emission maxima from 325 and 400 nm to 380 and 460 nm, respectively.

Falcipain-2 (50 nM) and falcipain-3 (300 nM) were assayed in triplicate at 25°C in 100 mM sodium acetate, 100 mM NaCl, 1 mM DTT, 1 mM EDTA, 0.01% Brij-35, 1% v/v DMSO, pH 5.5. Aliquots of 25 nmol (in 1 µl) from each of 20 sub-libraries of the P_1_-, P_2_-, P_3_-, and P_4_- libraries were added to the wells of a 96-well Microfluor-1 U-bottom plate (Dynex Technologies). The final concentration of each compound was 31.25 nM in 100 µl final reaction volume. The assays were initiated by addition of pre-activated enzyme and monitored fluorometrically for 30 min with a SpectraMax Gemini fluorescence spectrometer (Molecular Devices) with excitation at 380 nm, emission at 460 nm, and cutoff at 435 nm.

### Hydrolysis of globin peptides in the presence of H_2_^18^O

Synthetic peptides of 12 amino acid length scanning the entire sequences of α and β globin, with 4 amino acid overlaps between peptides, were custom-ordered from Sigma-Genosys. Each peptide (250 mM) was incubated in duplicate with 65 nM falcipain-2 or falcipain-3 in 25 mM sodium acetate, pH 5.5, 2 mM DTT, and 50% H_2_^18^O at 37°C. Reactions were started by adding enzyme after spotting the 0 minute time point. Aliquots were spotted after 0, 5, 10, 15, 20, 40, 60, 75, 90, 120, and 180 minutes, and overnight directly onto stainless steel MALDI-target plates (Applied Biosystems) and mixed with an equal volume of matrix (10 mg/ml sinapinic acid in 50% acetonitrile, 0.1% trifluoroacetic acid) which stopped the proteolytic reactions. MALDI-TOF MS/MS analysis was performed on a 4800 Proteomics Analyzer mass spectrometer (Applied Biosystems) in positive ion mode equipped with TOF/TOF optics and a 200 Hz NdYag laser. For collision-induced dissociation, the collision cell was floated at 1 kV, the resolution of the precursor ion selection was set to 150 FWHM, and air was used as the collision gas at 5×10^−7^ Torr.

### Analysis of peptide hydrolysis data

In the presence of H_2_^18^O, protease-peptide interactions can result in the incorporation of up to two ^18^O-atoms in carboxylate peptide products [19], [20]. The second incorporation, which depends on the ability of the enzyme to rebind the cleaved peptide product and catalyze the carbonyl oxygen exchange reaction, was rarely observed. Therefore, in the presence of 50% H_2_^18^O, peptides that were proteolytically generated displayed an ^18^O-isotope signature of 1∶1 (unlabeled: single labeled) at their newly-formed C-termini.

The extent of ^18^O incorporation for each peptide was determined by linear regression analysis of the raw spectra using least squares fitting with a model consisting of a linear combination of four components, the theoretical average in isotope clusters positioned at m/z, (m+2)/z and (m+4)/z, and a constant term. Linear regression computed four multipliers, the amplitude of the unlabeled, singly-labeled, and doubly-labeled peaks, and the baseline. It was thereby possible to measure the unlabeled, singly labeled, and doubly labeled contributions for each peptide identified in the mass spectrum.

We used the following criteria to positively identify new cleavage products: a) a new C-terminus had to be formed to allow ^18^O-incorporation; b) the single ^18^O-incorporation had to account for 50% of the entire isotopic envelope (±10% experimental error); c) peptide needed to be observed in replicate analyses and in consecutive time points (except for the overnight time point); and d) labeled peptide should not be detected at the zero time point, with the following exceptions: e) peptides that started off with >60% unlabeled fraction, with subsequent decrease in unlabeled peptide were included; f) peptides with initial single label, followed by double label were included; g) peptides with single label in a minority of time points slightly >60% were included; h) if the single label significantly increased with time, peptides with slight labeling at 0 min were included; and i) As expected, proteolytic products that were formed while the C-termini stayed intact did not incorporate ^18^O. Hence, these newly generated peptides with intact C-termini: 1) should display less than 20% labeling (accounting for experimental error); 2) these peptides should be seen in at least two time points and in replicate analyses (unless it is observed only in the overnight time point); and 3) these unlabeled peptides should not be seen at not at the zero time point.

### Hydrolysis of Human Hemoglobin

For analysis of hydrolysis of intact hemoglobin, reaction conditions and enzyme concentration were as described for experiments with 12-mer peptides, except that the substrate was 2.5 µM human hemoglobin (Sigma) and were incubated for 180 minutes or overnight. At the end of the incubation, peptides were separated by ultra-filtration (12,000×g for 40 min; Sartorius Vivaspin columns, nominal molecular weight limit 3,000 or 5,000 Daltons). Ultra-filtrates containing the cleavage products were analyzed by LC MS/MS on a QStar XL mass spectrometer (Applied Biosystems/Sciex) equipped with a nano 2DLC system (Eksigent) and a Nanospray II source (Applied Biosystems/Sciex). External calibration was performed in MS/MS mode using fragment ions of Glu-fibrinopeptide as references. An LC Packings PepMap C18 trap column (300 µm i.d., 5 mm length, 300 Å pore size, 5 µm particle size) and a column (75 µm i.d., 15 cm length) self-packed with Jupiter Proteo C12 end-capped material (90 Å pore size, 4 µm particle size) were used for desalting and reversed phase peptide separation, respectively. A 20 minute linear gradient from 2% B to 50% B was run at a 250 nL/min flow rate, utilizing solvents A: (2% acetonitrile/0.1% formic acid) and B (80% acetonitrile/0.08% formic acid). Precursor ion selection employed an automated routine that consisted of a one survey MS scan (1 s, m/z 400–1700) and two MS/MS scans (1 s, m/z 60–1500); nitrogen served as a collision gas and collision energy was automatically adjusted depending on the size and charge state of the precursor ion. Mass spectrometry data were analyzed using a laboratory information system that utilized Mascot Distiller software (Matrix Science) for spectral processing and peak detection. Peptides were identified by using the Mascot algorithm (Matrix Science; version 2.1) to search against human proteins in the SwissProt database via nonspecific digestion (*i.e.* “no enzyme” search). Identities of peptides were confirmed by manual interpretation of the MS/MS data.

## Results

### Expression of falcipain-2 and falcipain-3

Falcipain-2 and falcipain-3 were expressed in bacteria, purified, and refolded to active proteases. These proteases have previously been shown to readily hydrolyze hemoglobin [15], [17], and also to cleave peptide substrates after Leu-Arg-, Val-Leu-Arg-, and Val-Leu-Lys- [17]. These substrates were tested based on the known specificities of other papain family cysteine proteases [21]. However, more detailed characterization of the substrate specificity of these proteases has not been reported. To better characterize falcipain activities and determine whether a clear pathway of hemoglobin degradation by falcipains is present, activities were studied by three assays, evaluating action against a tetrapeptide library, 12-mer peptides representing the sequences of α and β globin, and intact hemoglobin. All assays were performed under conditions matching, to the best of our knowledge, those of the *P. falciparum* food vacuole, the site of hemoglobin hydrolysis by the falcipains, notably at pH 5.5.

### Hydrolysis of a positional scanning tetrapeptide library

**Figure 1:**
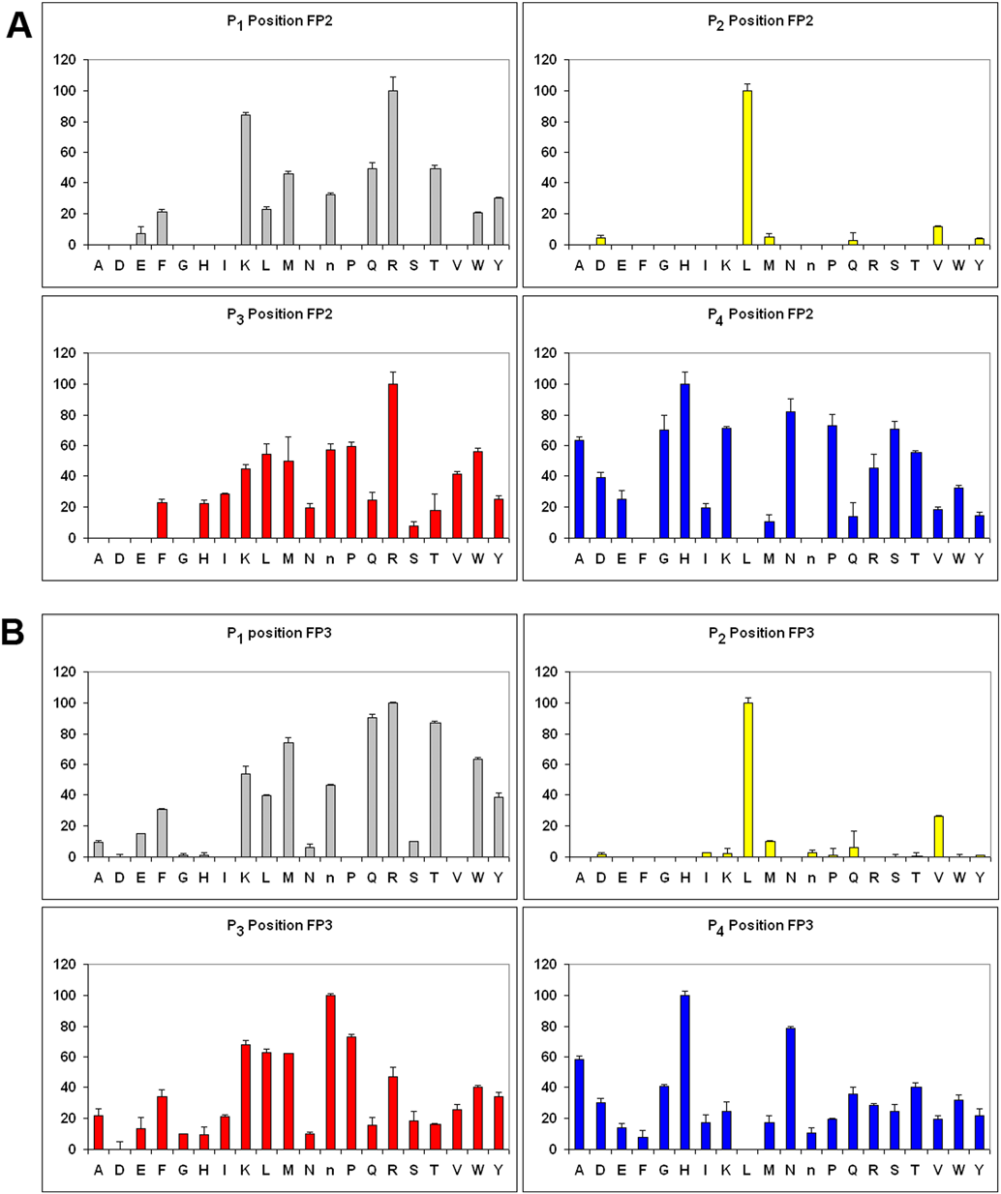
Cleavage preferences of falcipain-2 and falcipain-3 against a tetrapeptide library. P_1_ P_2_, P_3_ and P_4_ complete diverse libraries were used to determine specificities. Activities are displayed as percentage of the maximum for each position. Amino acids are represented by the single-letter code (n is for norleucine). Error bars represent standard deviations of results from three experiments.

To study the amino acid preferences of falcipain-2 and falcipain-3 at the P_1_ to P_4_ positions, we studied hydrolysis of positional scanning tetrapeptide libraries (Fig. 1). In each library, each tetrapeptide had a fixed amino acid at one position (P_1_, P_2_, P_3_, or P_4_) and randomized amino acids at the other positions [18]. P_1_ preferences for the two enzymes were similar. Both falcipain-2 and falcipain-3 showed a P_1_ preference for Arg, with activity also high against substrates with P_1_ Lys, Gln, Thr, and Met. Considering the P_2_ position, both falcipain-2 and falcipain-3 had a strong preference for Leu and a slight preference for Val. Amino acid preferences were less pronounced at the P_3_ and P_4_ positions, although specificities for the two enzymes were similar. At P_3_, positively charged amino acids, hydrophobic amino acids, and Pro were preferred. P_4_ His and Asn were preferred by both enzymes, with particularly low activity against peptides with hydrophobic amino acids at P_4_.

### Hydrolysis of globin peptides

**Figure 2:**
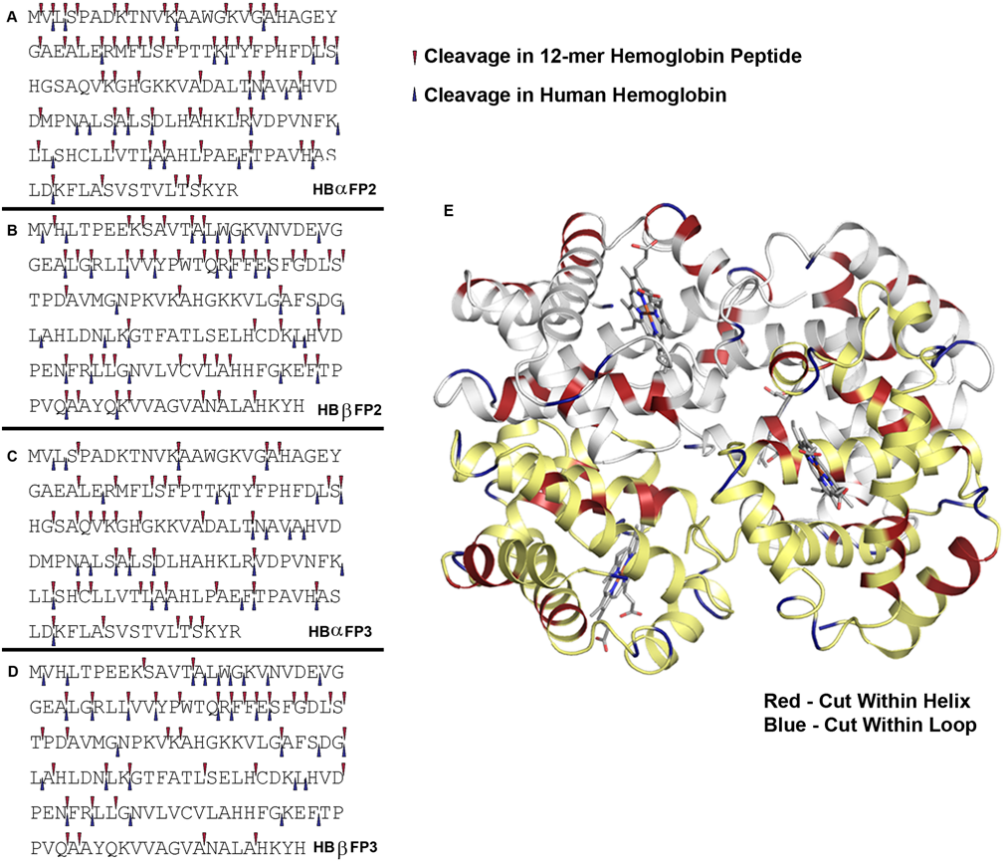
Hemoglobin cleavage sites for falcipain-2 and falcipain-3. A–D. Globin peptides and intact human hemoglobin. Cleavage sites for the two proteases based on analysis as described in Methods of hydrolysis of 12-mer peptides (top arrows, data from all time points) and intact hemoglobin (lower arrows, data from both 180 min and overnight time points) are shown for α and β globin. E. Intact human hemoglobin. The schematic shows α globin subunits in white and β globin subunits in yellow. P_1_ residues at cleavage sites within helices are in red and within loops are in blue.

**Figure 3:**
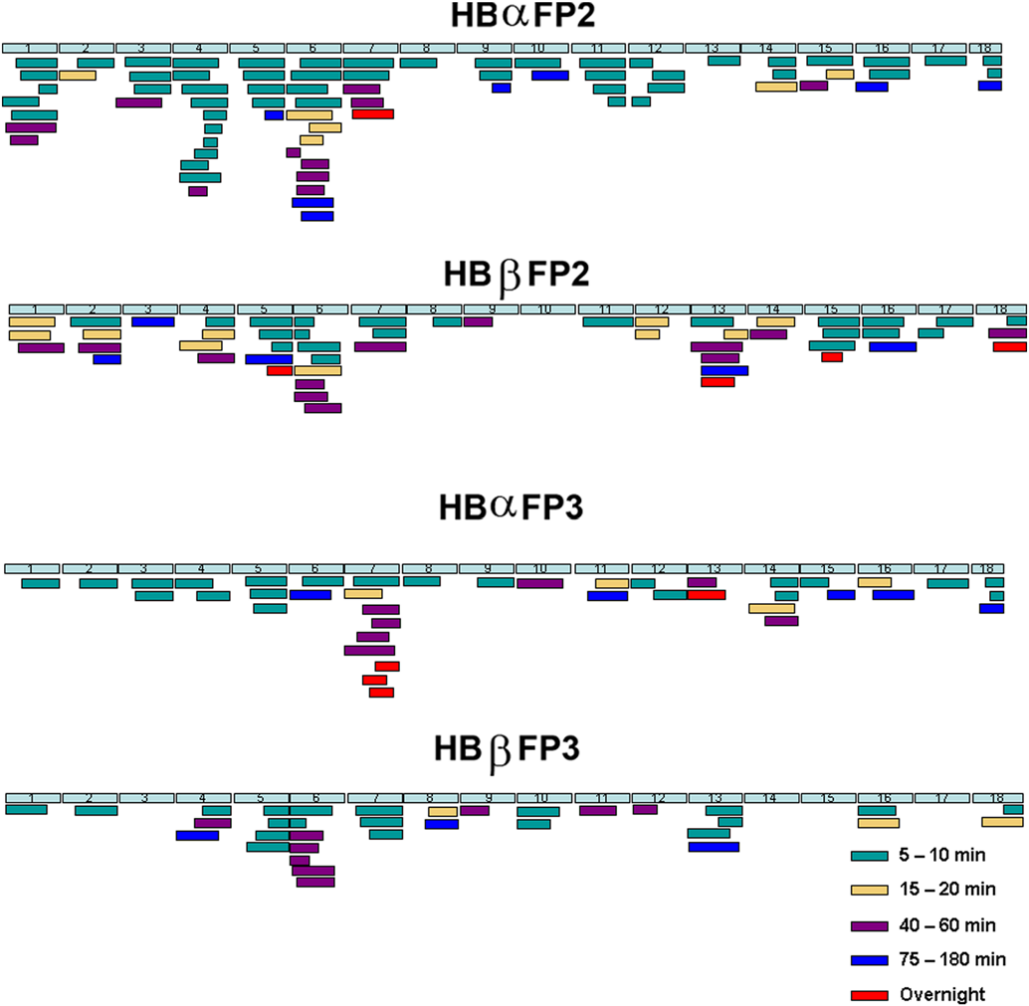
Time course for cleavage of globin peptides. Schematic representation of cleavage fragments over the indicated time points based on analysis of cleavage of 12-mer peptides spanning the sequences of α and β globin.

**Figure 4:**
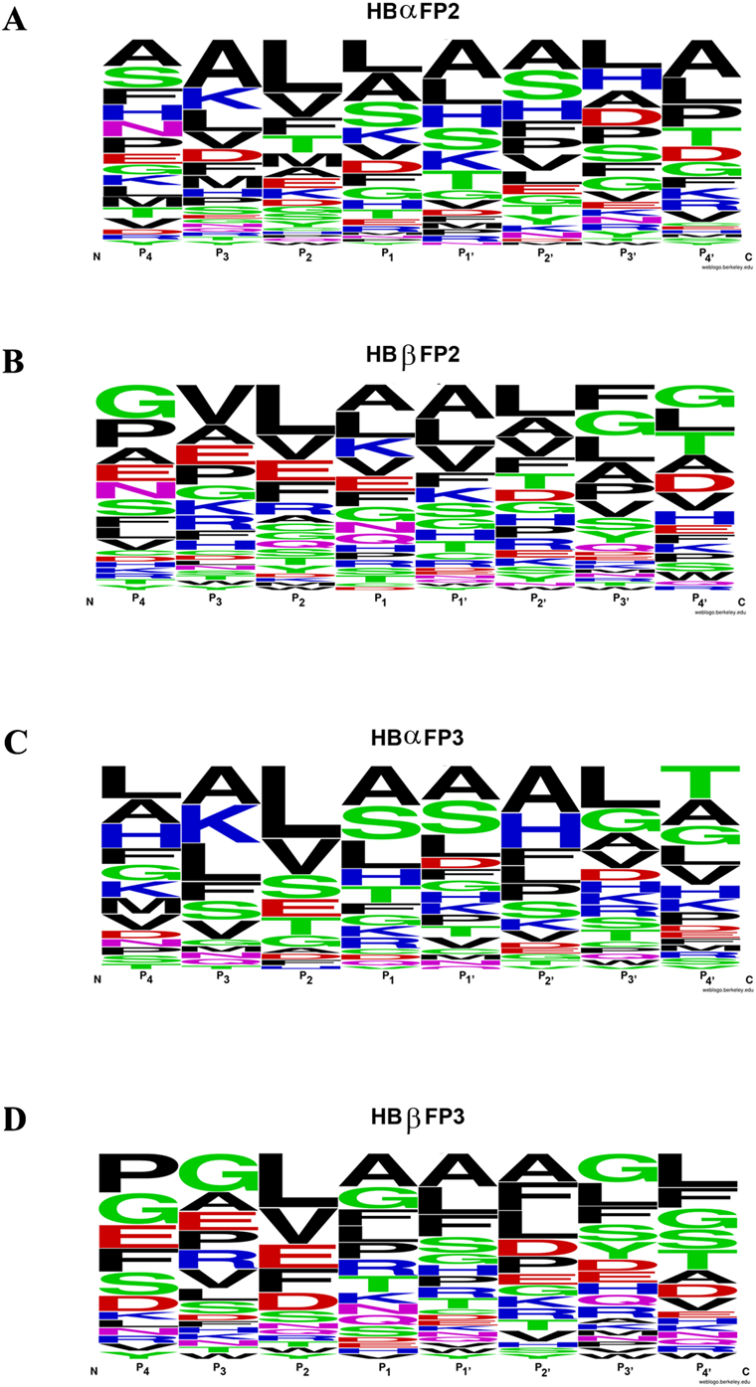
Cleavage site preferences for falcipain-2 and falcipain-3. Preferences for amino acids (single letter code) at P_1_-P_4_ and P_1_′-P_4_′ are shown schematically. At each position, the height of each amino acid corresponds to its frequency at cleavage sites based on analysis of α and β globin. WebLogo (weblogo.berkeley.edu), a free software for plotting consensus sequences, was used to plot P_4-_ P_4_′ preferences.

We next evaluated cleavage of 12-mer peptides spanning the sequences of α and β globin, with 4 amino acid overlaps such that each potential cleavage site was considered with at least 3 P or P′ amino acids present. To best distinguish falcipain cleavage products from minor fragments contaminating synthetic peptides, the proteases and peptide substrates were incubated with 50% H_2_^18^O. With proteolytic cleavage, the C-terminal ends of the newly generated peptides, but not false products, bore ^18^O either by enzyme catalysis or isotope exchange [19], [20]. Peptides were incubated with falcipain-2 or falcipain-3 for varied intervals, and reaction products were assessed by MALDI-TOF MS/MS analysis. We used stringent criteria to distinguish newly generated peptides from false positive and ambiguous products, based on ratios of labeled to unlabeled peptides and frequencies of appearance at several time points. Multiple cleavage sites were identified for both falcipains (Fig. 2a–d, top arrows). Considering the sequences most preferred in our positional scanning library analysis, cleavages by falcipain-2 were seen at 25 of 36 positions with P_2_ Leu and by falcipain-3 at 22 of 36 such positions. Analysis of the step-wise hydrolysis of peptides over time yielded a complex picture for both α and β globin and either falcipain-2 or falcipain-3 (Fig. 3). Identification of the most common amino acids at cleavage sites was, of course, influenced by the prevalence of different residues in hemoglobin. At the P_2_ position, Leu was most common, but cleavages of peptides with P_2_ Val and other amino acids were also well represented (Fig. 4). A more thorough analysis of cleavage for each amino acid at each P_1–4_ position is in supplementary material (Table S1; Figure S1). These experiments also showed that the most preferred P_1_′ amino acids were Ala and Leu. However, this preference was modest, with cleavages demonstrated at sites with multiple different prime-side amino acids. At the P_2_′ position Ala was slightly preferred. There was no particular amino acid preference at the P_3_′ or P_4_′ position.

### Hydrolysis of intact human hemoglobin

For our third analysis, we assessed hydrolysis of full-length hemoglobin by falcipain-2 and falcipain-3. A simple step-wise pathway for hemoglobin degradation was not seen. Rather, we identified over a hundred peptides of molecular weight 9 kDa or less after 5 minute incubations of human hemoglobin with falcipain-2 or falcipain-3 (data not shown). For simplicity, we therefore concentrated on identification of hemoglobin cleavage fragments after an extended incubation with falcipain-2 or falcipain-3. Reactions were performed after incubation of hemoglobin with 50% H_2_^18^O, and the identities of hemoglobin cleavage fragments were characterized by LC-MS/MS. Data were filtered based on ^18^O incorporation, as described above for analysis of peptide hydrolysis. We then mapped cleavage sites on the sequences of α and β globin (Fig. 2a–d, bottom arrows). Cleavage sites were identified in both the loops (blue color) and helices (red color) of human globin, with no particular preference for buried or exposed cleavage sites on the hemoglobin tertiary structure (Fig. 2e). Of note, the data obtained for falcipain-2 were nearly identical to those for falcipain-3 for hydrolysis of hemoglobin and were very similar to those for hydrolysis of globin peptides by both proteases.

## Discussion

Falcipain-2 and falcipain-3 are critical hemoglobinases of *P. falciparum*. These enzymes have been extensively studied, with characterization of kinetic properties [15], [17], confirmation of key functions with inhibitor [22], [23] and gene disruption studies [10], [11], and identification of unique motifs that are required for protein folding [24], [25], interaction with hemoglobin [26], enzyme structure [27], [28], [29], and targeting to the parasite food vacuole [30], [31]. However, characterization of a specific pathway for hemoglobin hydrolysis by falcipain-2 and falcipain-3 has been difficult. We now present a detailed analysis of hydrolysis of short peptide substrates, 12-mer peptides encompassing α and β globin, and intact human hemoglobin by falcipain-2 and falcipain-3. We show that the proteases have distinct specificity for short peptide substrates with Leu at the P_2_ position, but that against larger substrates specificity is less marked. Indeed, the proteases rapidly cleaved globin peptides and intact hemoglobin at multiple different sequences. These results suggest that falcipain-2 and falcipain-3 do not cleave host hemoglobin via an ordered process, but rather that the proteases efficiently cleave the substrate by rapid hydrolysis at many different sites on the protein.

We evaluated hydrolysis of small peptide combinatorial libraries by falcipains, a standard technique. Other proteases that have been evaluated in this manner include caspases [32], the serine protease granzyme b [32], [33], [34], a coronavirus cysteine protease [35], a tick asparaginyl endopeptidase [36], and human kalikreins [37]. Although this methodology is most useful for highly selective enzymes, valuable information can also be extracted from substrate profiles with enzymes that are quite promiscuous, as in the case of the falcipains and other papain family cysteine proteases [21]. Of note, we observed marked selectivity in P_2_ preference for falcipains using small peptide substrates. Hydrophobic amino acids at P_2_ were strongly preferered by falcipain-2 and falcipain-3, as seen with many other papain-family proteases [21]. However, the specific preference for a single amino acid, Leu, at P_2_, was more striking than the case for any of the 8 other papain-family proteases recently studied in the same manner [18]. Of the 8 other proteases, the next most specific enzyme was papain, with about 5-fold greater activity for peptides with Val at P_2_ than any other peptides. Other papain-family proteases not included in the prior survey appear to show a stronger preference for P_2_ Leu, notably the human proteases cathepsin K and cathepsin S [21]. As discussed in more detail below, we found that the striking preference for P_2_ Leu in small substrates did not predict specificity only for cleavage sites with P_2_ Leu in larger peptide or protein substrates. Nonetheless, this specificity has been seen also in small molecule inhibitors of falcipain-2 and falcipain-3; inhibitors with either Phe or Leu at P_2_ were effective, but typically activity improved ∼10-fold for fluoromethyl ketones, vinyl sulfones, and aldehydes with P_2_ Leu [23], [38], [39]. Thus, although of limited utility in discriminating activity against native protein substrates, the elaboration of enzyme specificity using tetrapeptide scanning libraries provided accurate prediction of optimal small molecule inhibitors. Appreciation of the fine specificity of inhibition of falcipains is contributing to drug discovery efforts directed against these proteases [6].

Our experimental conditions for globin peptides and native hemoglobin were slightly modified from a previous study [14], so that they were suited for ^18^O incorporation and subsequent mass spectrometry. These conditions were designed to match those in the intact food vacuole, including modest reducing conditions (1 mM DTT) and pH of 5.5. Prior studies have estimated the pH of the food vacuole at 4.5–5.8 [40], [41], [42], [43] and shown the pH optima of falcipain-2 and falcipain-3 to be ∼4.5–6.0 [15], [17]. The enzyme∶substrate ratio was slightly higher than that used previously [14]; these conditions gave us optimum results with substrate degradation and ^18^O incorporation, which is also an enzyme-dependent process.

Ordered pathways for the hydrolysis of hemoglobin have been demonstrated in studies with isolated aspartic proteases from two parasites that hydrolyze this substrate, schistosome worms that degrade hemoglobin extracellularly [44] and malaria parasites that degrade hemoglobin in an intracellular acidic organelle [12]. However, prior attempts to map out pathways of hemoglobin hydrolysis for cysteine proteases of schistosomes or malaria parasites have been unsuccessful due to an inability to identify the ordered generation of cleavage fragments (Shenai BR, Sajid M, and Rosenthal PJ, unpublished observations). A key goal of the present study was to more rigorously test the hypothesis that falcipain-2 and falcipain-3 indeed hydrolyze hemoglobin through an ordered pathway of cleavage events and, in any event, to identify globin peptide and hemoglobin cleavage fragments after incubation with falcipains. Despite rigorous filtering to identify only bona fide cleavage fragments, we found that falcipain-2 and falcipain-3 cleaved hemoglobin simultaneously (within our limits of analysis) at many sites. Thus, the enzymes appear to degrade hemoglobin not via an ordered hydrolytic pathway, but through rapid hydrolysis of multiple sites. This result is consistent with known features of other papain-family cysteine proteases, which also have quite broad specificity against protein substrates [21]. Indeed, the prototype enzyme papain is used in a number of industrial applications that take advantage of its broad substrate specificity. Lysosomal cysteine proteases similarly demonstrate broad specificity, and appear to be responsible for extensive proteolytic activity in this organelle [21]. In malaria parasites, where the food vacuole appears to serve as the homolog of the lysosome, and where hemoglobin appears to be the primary proteolytic substrate [12], it seems likely that falcipain-2 and falcipain-3 are responsible for the bulk of cleavages that reduce hemoglobin from a complex tetramer to single amino acids. Additional proteases, notably plasmepsin I–IV [12], [45], dipeptidyl aminopeptidase I [46], [47] and falcilysin [48], [49], also appear to contribute to the efficient hydrolysis of hemoglobin in the food vacuole, and the presence of multiple proteases of multiple classes suggests a degree of redundancy in this process [9]. Despite the presence of multiple proteases in the food vacuole, it is noteworthy that treatment of cultured parasites with specific inhibitors of cysteine proteases fully blocked the hydrolysis of hemoglobin, highlighting the key role for the falcipains in this process [22]. Knockout of plasmepsins I–IV, the 4 known food vacuole plasmepsins, was not lethal to *P. falciparum* (although parasites with knockout of all four food vacuole plasmepsins demonstrated abnormal development) [50], [51]. In contrast, knockout of either falcipain-3 [11] or dipeptidyl aminopeptidase I [46] appears to be lethal, arguing for essential functions for these enzymes, although it is not known if the essential functions are hydrolysis of hemoglobin. Of note, it has recently also been shown that the activation of food vacuole plasmepsins is primarily mediated by falcipains, offering further evidence of a primary role for falcipains in mediating the hydrolysis of hemoglobin by erythrocytic *P. falciparum* parasites [52].

Results showing potent hydrolysis of hemoglobin by both falcipain-2 and falcipain-3 [14 and this report] and the localization of these falcipains in the food vacuole [7], [30] argue that a key function of these proteases is hemoglobin hydrolysis. However, it remains possible that they also perform other key functions, including the processing of plasmepsins (as discussed above) and possibly other food vacuole proteases [52] and the hydrolysis of erythrocyte cytoskeletal proteins to facilitate erythrocyte rupture [53], [54], [55]. As falcipain-2 and falcipain-3 are not biochemically identical and as they are expressed sequentially during the parasite life cycle, they may have non-overlapping biological roles, explaining in part the redundancy of papain-family proteases in *P. falciparum*.

Of interest, falcipain-2 and falcipain-3 contain a short C-terminal insertion that is unique among studied papain-family proteases and mediates interaction between the proteases and hemoglobin independent of the enzyme active site [29]. Removal of the insertion from falcipain-2 rendered a protease that remained active against peptide substrates, but could not hydrolyze hemoglobin [26]. This feature of the falcipains likely facilitates particularly efficient hydrolysis of hemoglobin and may contribute to the ability of the proteases to cleave hemoglobin at multiple sites. However, it is difficult to predict interaction between the protease and substrate based on known structures [28], [29], as once initial cleavages of hemoglobin take place, the structure of hemoglobin will be altered, and multiple peptide bonds will presumably be accessible to cleavage by the falcipains.

A prior study identified hemoglobin cleavage fragments after incubation of this protein with crude *P. falciparum* food vacuole extracts [56]. This analysis identified a series of hemoglobin cleavage fragments, with cleavage sites at mean intervals of 8.4 amino acids. Comparison of identified cleavage sites from the prior analysis of food vacuole extracts with our analysis of cleavage by recombinant falcipains identified many shared cleavage sites, but also cleavages identified only after incubation with food vacuole extract or only after incubation with recombinant falcipains. Specifically, only 15 of 37 cleavages identified in the prior study after incubation with food vacuole extracts were seen after incubation with falcipain-2 or falcipain-3. Therefore, some hemoglobin cleavages appeared to be generated uniquely by proteases other than the falcipains. However, considering both our globin peptides and intact hemoglobin results, we observed over 100 different cleavage sites within hemoglobin, with 90 of these not seen after incubation with food vacuole extract. Some falcipain activity may have been missed in studies of vacuole extracts due to chosen assay conditions, the presence of enzyme inhibitors in extracts, or loss of enzymes during sample preparation. In any event, our new results suggest that, although a diverse set of proteases is likely required for optimal hydrolysis of hemoglobin, the sequential expression of falcipain-2 in early trophozoites and then falcipain-3 in late trophozoites and schizonts [7] provides potent hemoglobinolytic activity for erythrocytic *P. falciparum* parasites.

Our study has important limitations. First, the precise analysis of cleavage of globin peptides or hemoglobin by falcipains proved to be technically very challenging. Analysis of products after incubations of globin peptides with the proteases identified many products, suggesting the presence of contaminating fragments in commercial peptides. We therefore used ^18^O labeling and assessments for reproducibility over time to identify genuine cleavage fragments. However, this approach necessarily involved the establishment of arbitrary filtering criteria, and may have inappropriately excluded some cleavage sites and/or included false cleavage sites. Second, analysis of cleavage of intact hemoglobin was cost- and labor-intensive, limiting our ability to evaluate a broad range of hydrolytic time points. Thus, our ability to identify any single hemoglobin cleavage site for falcipain-2 or falcipain-3 from our studies is somewhat limited, although cleavages seen consistently with both falcipains at multiple time points and with both peptide and intact hemoglobin substrates are very likely to represent true biological cleavage sites. Third, our study used ultrafiltration to isolate peptide cleavage fragments generated from intact hemoglobin. This procedure might lead to selective loss of certain peptides. To investigate that possibility, we previously analyzed tryptic digests of proteins prior to and after ultrafiltration, and we did not observe significant differences between the samples (Hardt, M., unpublished observation). Nonetheless, we cannot exclude the possibility that certain peptides might have been lost due to this separation technology, but, based on our overall sequence coverage of hemoglobin, this potential loss appeared to be minimal. In any event, our overall result is clear: falcipain-2 and falcipain-3 are capable of rapid hydrolysis of hemoglobin at multiple cleavage sites.

It is of interest that two common polymorphisms leading to alterations in the sequence of hemoglobin, HbS and HbC, offer protection against severe malaria [57], [58], [59], [60], [61], and many other polymorphisms in hemoglobin may offer protection against malaria. It is possible that this protection is mediated, in part, by altered susceptibility of mutant hemoglobins to hydrolysis by parasite proteases, including falcipains. This will be an interesting area for future study.

In summary, we have characterized the proteolytic specificity of two key cysteine protease hemoglobinases of the malaria parasite *P. falciparum*. Both falcipain-2 and falcipain-3, which are expressed sequentially, contribute importantly to the necessary hydrolysis of hemoglobin by intraerythrocytic parasites. Indeed, our results show that the falcipains rapidly hydrolyze hemoglobin at multiple sites, allowing efficient degradation of this substrate, and suggesting that the falcipains are the key proteases responsible for the massive hydrolysis of hemoglobin that occurs over the course of the intraerythrocytic life cycle of *P. falciparum*.

## Supporting Information

Figure S1 Graphical representation of amino acid preferences at P1–P4. P1–P4 amino acid preferences were plotted as a percentage of total cleavages, alongside percentage occurrence of each amino acid in the primary sequences of α and β globin.
(0.51 MB TIF)

Table S1 This table shows (in two separate worksheets) sequences of the precursor peptide (for 12mer α and β globin peptides) and peptide products obtained as a result of falcipain mediated proteolysis.
(0.05 MB XLS)
