# Supplementary figures and images for: Hemoglobin Cleavage Site-Specificity of the Plasmodium falciparum Cysteine Proteases Falcipain-2 and Falcipain-3
Source: PLoS One. 2009 Apr 9;4(4):e5156. doi: 10.1371/journal.pone.0005156 (PMC2663817; doi:10.1371/journal.pone.0005156)

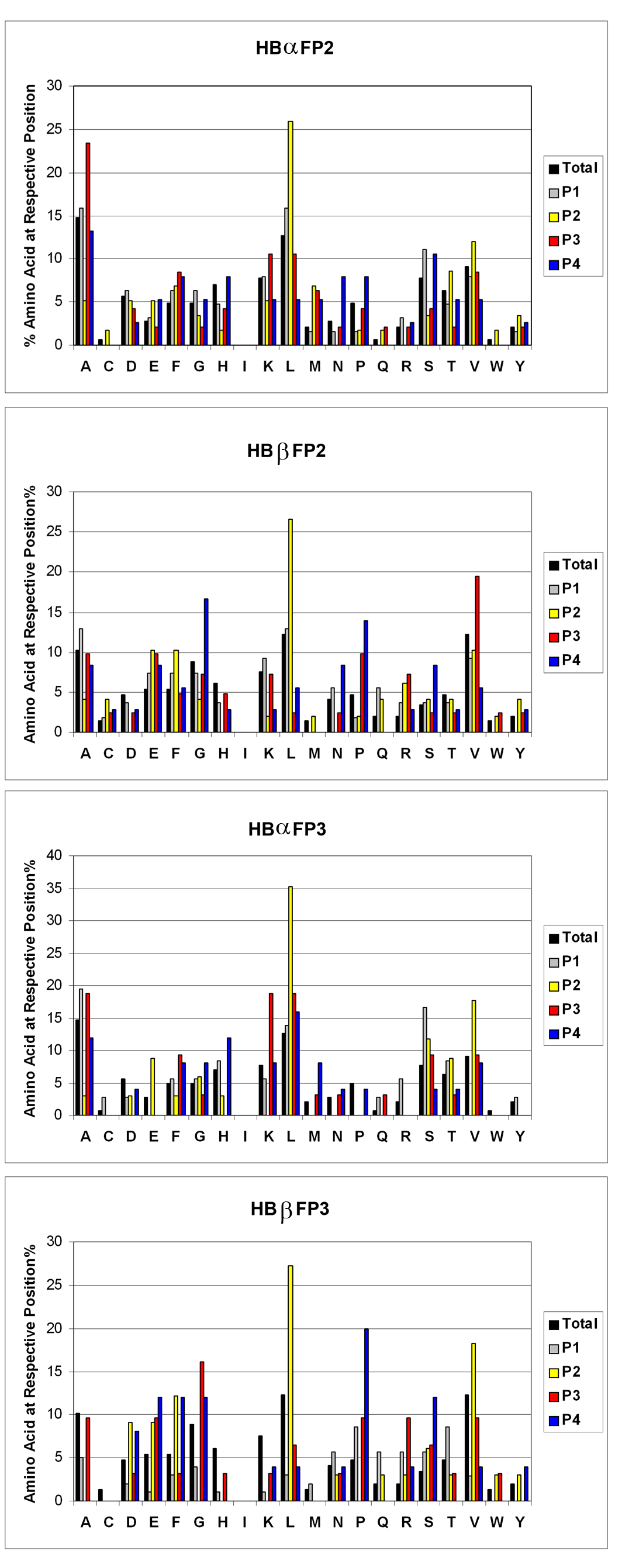

Supplement: Figure S1 — Graphical representation of amino acid preferences at P1–P4. P1–P4 amino acid preferences were plotted as a percentage of total cleavages, alongside percentage occurrence of each amino acid in the primary sequences of α and β globin. (0.51 MB TIF) [file pone.0005156.s001.tif]
